# Recent Advances in Cochlear Implant Electrode Array Design Parameters

**DOI:** 10.3390/mi13071081

**Published:** 2022-07-08

**Authors:** Yavuz Nuri Ertas, Derya Ozpolat, Saime Nur Karasu, Nureddin Ashammakhi

**Affiliations:** 1 Department of Biomedical Engineering, Erciyes University, Kayseri 38039, Turkey; deryabetul79@gmail.com (D.O.); karasusaimenur@gmail.com (S.N.K.); 2 ERNAM—Nanotechnology Research and Application Center, Erciyes University, Kayseri 38039, Turkey; 3 Institute for Quantitative Health Science and Engineering (IQ) and Department of Biomedical Engineering (BME), Michigan State University, East Lansing, MI 48824, USA

**Keywords:** cochlear implant, electrode, spiral ganglion cells, cochlea, stimulation

## Abstract

Cochlear implants are neural implant devices that aim to restore hearing in patients with severe sensorineural hearing impairment. Here, the main goal is to successfully place the electrode array in the cochlea to stimulate the auditory nerves through bypassing damaged hair cells. Several electrode and electrode array parameters affect the success of this technique, but, undoubtedly, the most important one is related to electrodes, which are used for nerve stimulation. In this paper, we provide a comprehensive resource on the electrodes currently being used in cochlear implant devices. Electrode materials, shape, and the effect of spacing between electrodes on the stimulation, stiffness, and flexibility of electrode-carrying arrays are discussed. The use of sensors and the electrical, mechanical, and electrochemical properties of electrode arrays are examined. A large library of preferred electrodes is reviewed, and recent progress in electrode design parameters is analyzed. Finally, the limitations and challenges of the current technology are discussed along with a proposal of future directions in the field.

## 1. Introduction

The use of neuromodulation implants and bioelectronic devices has been expanding rapidly and is expected to lead to a have a major impact on the practice of medicine [1,2]. Cochlear implants (CI) are considered to be the most effective treatment for patients experiencing severe sensorineural hearing loss, and the current number of CI recipients in the world is more than 400,000 [3]. Hearing via CI operation starts with the sound waves hitting a microphone, which is placed on the skull [4]. The microphone detects the incoming sound and relays it to a processor, where it is filtered, processed, digitally encoded into a radio frequency (RF) signal, and transmitted to the inner part of the implant. The receiver decodes the RF signals into electrical currents and transmits them to the electrodes. Finally, the electrodes stimulate auditory nerves in the ear and enable the brain to perceive the sound [5,6]. Electrical stimulation of the CI electrodes creates an electric field that propagates along the cochlea, affects multiple electrodes, and causes stimulation of not only the nerves at the electrode site of interest but also the nerves at the electrode populations around the site of interest [7]. It has been shown that responses generated by superimposing the electrical signals evoked from different electrodes can change the information provided by separate channels, which may reduce the selectivity and the number of effective channels in CI. Therefore, preferred electrode organization in CI is an important parameter that determines hearing quality. CIs are based on the use of electrodes commonly made of platinum-iridium (Pt-Ir) [8] and silicon carriers which hold electrodes and electrode wires [9]. However, current CIs have limitations regarding the stiffness of the electrode arrays, having an insufficient number of electrodes, and providing a sub-optimal hearing experience [10,11]. Recent work on improving these concerns enabled CI designs that can provide a personalized sound experience [12,13,14]. Although electrode arrays and electrode materials vary between different designs, electrode shapes remain the same, mainly being square, rectangular, and spherical [9]. On the other hand, active research is being pursued to discover new electrode array materials as alternatives to the commonly used silicon. Although polymer-based alloys and polymers were reported to be good candidates [15,16], an electrode array that can fully provide high biocompatibility, flexibility, a dielectric constant, biodegradability, and processability has not yet been demonstrated [17]. The distance between electrodes is an important factor that affects channel interaction. When the electric field of stimulating electrodes spreads over neighboring nerve population, field overlap occurs, which decreases speech recognition [18]. To achieve better hearing, a larger distance between electrodes is required [19].

Current CIs have three types of stimulation modes: monopolar, bipolar, and tripolar [20,21,22], each of which having pros and cons. For example, in the monopolar stimulation mode, an electric field is generated between widely spaced intracochlear and extracochlear electrodes, where current flows from these electrodes and stimulates larger nerve populations. However, in the bipolar stimulation mode, an electric field occurs between two adjacent active and reference electrodes and stimulates smaller nerve populations [23,24]. In this regard, while monopolar stimulation generates the greatest overlap of electric fields, bipolar stimulation increases channel independence while reducing the current spread [25]. During surgical implantation, real-time observation of the electrode array is not possible. Recently, sensors placed into/on electrode arrays have been proposed to spatially track the array and sense the tip pressure [26], which may prevent surgical trauma and offer implantation procedure, comfortable for surgeons and patients.

There have been several review papers focusing on the hearing quality of CI patients, electrode tip properties, surface patterning of electrodes, and other clinical aspects [27,28,29,30,31], yet a review addressing the recent developments in CI electrodes which discusses electrode materials, shape, electrode arrays, and their mechanical properties along with the incorporation of sensors to electrode arrays is lacking. This paper discusses the current status and challenges of CI technology and proposes future directions toward which the field is progressing. It includes an in-depth discussion of the electrodes used in CIs, specifically, electrode materials and geometric shapes, the effect of the distance between the electrodes on the nerve stimulation performance, electrode types, stiffness and flexibility of electrode arrays, and sensors which are embedded in electrode arrays. Recent advances in each of these aspects are summarized, and suggestions to improve the current drawbacks are provided.

## 2. Electrode and Electrode Array Design

### 2.1. Electrode Materials

Electrode materials used for neural stimulation have varied for many years. Biocompatibility, high charge capacitance, high conductivity, and stability are the main qualities desired in CI electrode materials. The most popular alloy materials for CI electrodes are titanium-nitrite, titanium (Ti)-Ir, Ti-tantalum, iridium-oxide, and Pt-Ir [32]. In addition, although rare, polymer-coated metal electrodes are among the studied materials. However, the development of cochlear implant electrodes continues in electrode arrays. The main goal of this technological development is to produce an electrode array that is close to the spiral ganglion cells and can perfectly be wrapped around the modiolus, because the electrode in the cochlea should be able to embrace the modiolus to reduce the distance between the targeted spiral ganglion cells and reduce the power consumption and channel interference [33,34,35,36]. Here, minimizing channel interference can provide users with a better hearing experience. Additionally, softer and more flexible electrode arrays are required to reduce the insertion trauma of the electrode array and postoperative fibrous tissue formation [37]. A hydrogel-based electrode array that can be bent-flexible when exposed to a saline solution (simulating the intracochlear fluid perilymph) was fabricated [38]. Here, a reliable hydrogel-driven self-bending CI electrode array was realized with a dummy electrode carrier made of silicon rubber and carbon nanotubes. Trauma during the electrode placement can cause permanent hearing damage. The localized administration of steroid drugs is important to minimize hearing damage. An electrode array consisting of a microfabricated flexible electrode array and a three-dimensional (3D) microscaffold for steroid release was fabricated [39]. Threshold shifts, which refer to better hearing quality, tended to be lower in the group in which steroid-containing microscaffold cochlear electrode array (MiSCEAs) were implanted. It was proposed that the feasibility of the 3D MiSCEA will enable the development of a potential next-generation cochlear electrodes with improved steroid release dynamics. Patients having a cochlear implant may lose residual hearing at low frequencies within a few months after cochlear implantation [40]. There are two important issues in this respect: (1) inflammatory response caused by mechanical trauma, and (2) fibrosis and new bone formation in the cochlea, which increase the impedance of the electrodes and compromised neuronal activity [41,42,43,44,45,46,47]. Dexamethasone release from poly-ε-caprolactone (PCL) coating of cochlear electrodes was used to control cochlear fibrosis caused by cochlear implantation [48]. An ideal coating should have certain level of thickness to hold enough drugs without impacting the performance of electrodes, and the drug release should be prolonged to have an effective long-term treatment. Implantation experiments in rats showed that drug-loaded PCL coating of electrodes could reduce surgery-induced inflammation.

**Figure 1:**
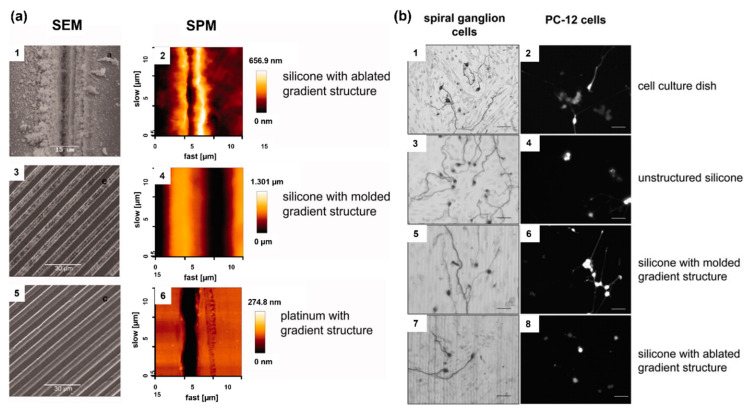
(**a**) Assessment of the various microstructures on electrode materials with scanning electrode microscopy (SEM) and scanning probe microscopy (SPM); ablated silicone elastomer (1,2), molded silicone elastomer (3,4), and platinum sputtered glass (5,6). (**b**) Microstructure-guided neurite outgrowth of spiral ganglion cells (left) and PC−12 cells (right) on the cell culture dish as the control (1,2) as well as on unstructured silicone (3,4), silicone molded gradient microstructure (5,6), silicone with ablated gradient microstructure (7,8). Bars: 50 μm. Reprinted with permission from [50]. Copyright 2012, Wiley.

During the first few weeks after the implantation of a CI electrode array, electrical impedance at the electrode–tissue interface increases due to the formation of fibrous tissue around the electrode array, which diminishes the interaction between the electrodes and target tissue. Therefore, it is of great clinical interest to modify electrode carrier material to improve the electrode–nerve interface. While fibrous tissue growth needs to be reduced to prevent electrode array encapsulation, the electrode carrier material should also not compromise the interaction with neuronal cells. To improve the electrode–nerve interface in these patients, it is therefore aimed to reduce fibrous tissue formation around electrode arrays after implantation. The impedance of CI electrodes can be lowered with the technologies developed on the electrode array, specifically by utilizing micro-nano structures formed on the electrode surface. Nanostructures were formed on the electrode surfaces, and surface-structured electrode arrays were implanted in guinea pigs. Compared to the control group, surface structured electrodes induced lower impedance, showing the potential of a nanopatterning approach [49]. Micropatterning of electrode surfaces can improve the interaction between electrode and neuronal cells because electrode contacts need to be free of fibrous tissue for effective stimulation, and it is desired to guide and attach neurons or their extensions to the electrodes. Electrodes with different surface patterns were produced, and their interactions with spiral ganglion cells and PC-12 neuronal-like cells were investigated (Figure 1). Both cell types were aligned parallel to the microstructures of both silicone and Pt surfaces, indicating that the microstructure induced the guidance of neurites, which also lowers fibroblast growth on the electrodes [50].

Implants generally induce fibrous tissue formation [51,52]. Coated electrodes can both help in the delivery drugs to the apical parts of the cochlea and the prevention of the formation of fibrous tissue. The main purpose in electrode coating has been to minimize the fibrous tissue response [53]. CI electrodes can also be produced using more than one metal. Pt is doped with Ir in order to slow down the wear rate standout [54]. Carbon nanotubes (CNTs) were used due to their suitable mechanical properties; however, the electrical conductivity was far lower than that of Pt [55]. Recently, natural polymers have also been used in CI electrode coating studies. Alginate coating of the electrodes provided high biocompatibility and stability, encouraging future studies on this subject [56]. Thus, coating and micropatterning of CI electrodes represent an active area of research in the field to improve the performance of Pt and Ir electrodes by reducing the impedance and noise, increasing signal-to-noise ratio, and preventing or reducing the formation of fibrous tissue.

### 2.2. Electrode Shape

**Figure 2:**
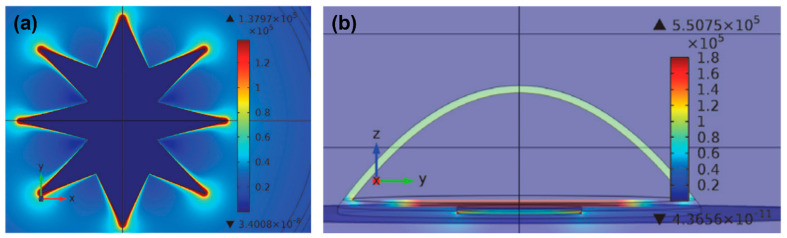
(**a**) Current density distribution on a star electrode upon −60 mV/ms of falling voltage ramp stimuli at t = 10 ms. (**b**) Electric field at different areas of the cell at the termination of the stimulus voltage ramp on the circular electrode. Reprinted from [59]. Copyright 2013 according to Attribution-NonCommercial-NoDerivatives 4.0 International (CC BY-NC-ND 4.0), which allows to copy and redistribute the material in any medium or format.

Electrodes used in CI devices represent an approach to the realization of neural stimulation, and the geometric design of the electrodes is vitally important for nerve stimulation. However, there are limited studies on this subject. The common electrode geometry for many years has been rectangular or square in shape. Rectangular electrodes are preferred in almost all models by Advanced Bionics and MED-EL manufacturers. In the first years of CI production, ring-shaped electrode designs were developed. The biggest nuance about the electrode geometry is that electrode surface should be large enough to excite without affecting the electromagnetic fields of the nearby electrodes, hence preventing unwanted stimulation. In recent years, innovative studies on this subject have been published [57,58]. In the original electrode design, an octagonal star with pointed corners was used in order to achieve a higher electric field and sealing resistance [59]. Figure 2 shows the electric field generated by a star electrode and a commonly used circular electrode. The high electric field created by the eight-pointed star electrode was about three times larger than the spherical design. Another study reported high-peripheral electrodes, which have the same surface area, yet the circumference of each one of them was 2–4 times larger than that of planar electrodes with a circular shape [60]. Unlike electrodes with regular circumference, planar electrodes with irregular (serpentine) circumference were found to be more efficient in activating axons located farther from them and reduced power consumption by ~10%. Various computational experimental models have been developed to determine the efficiency of electrodes. The aim here is to enhance the electrical energy transmitted to the auditory nerves, thereby decreasing wasted energy, and increasing the efficiency and effectiveness of the CI system. The current distribution of the human cochlea was determined during CI electrical stimulation using finite element (FE) analysis [61]. The efficiency of the electrodes was assessed by applying genetic algorithms along with computational models and FE analysis to optimize the shape and dimensions of the CI electrode array. There are still not enough studies on the effect of electrode geometry on neural stimulation devices. Future research on more comprehensive studies of the electrode surface and electric fields should improve the CI design accordingly.

### 2.3. Effect of Electrode Spacing on Stimulation of Spiral Ganglion Cells

**Figure 3:**
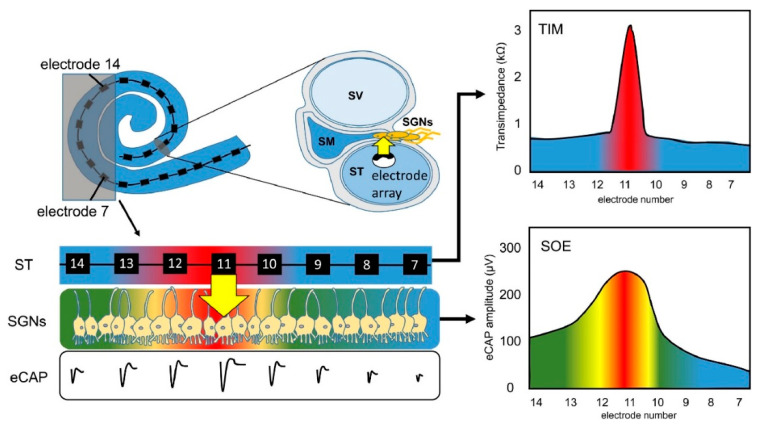
Principle of spread of neural excitation (SOE). Stimulation of electrode 11 spreads over neighboring electrodes and causes overlapping of excitation on spiral ganglion neurons (Scala tympani, ST; electrically evoked compound action potential, eCAP; spiral ganglion neurons, SGNs; transimpedance matrix, TIM). Reprinted with permission from [67]. Copyright 2021, Elsevier.

When sound hits the microphone, the processor receives it, amplifies it, and separates it into different frequencies with band-pass filters [6,62]. This band-pass filtered information is sent to the inner part of the CI, where the receiver decodes the band-filtered RF signals and sends current to the electrode for the stimulation of nerves [63]. The current generated from filtered information flows between the active and return electrodes of the CI, and the configuration of these electrodes defines channels of the choclear implant [64]. Each channel has a defined band-pass filter [65]. The transmission efficiency of electrical stimulation depends on the spacing of electrodes, stimulation modes, surface of electrodes, and distance between the electrodes and nerves [66]. However, channel interaction due to broad spread of excitation is an inherent problem of multielectrode stimulation [18]. CI users suffer from insufficient spectral resolution due to channel interaction. Theoretically, an electrode stimulates the area directly facing it, but the electrical field generated by one stimulating electrode can cause the stimulation of neighboring spiral ganglion neurons [67,68]. Figure 3 shows this phenomenon, which is known as the spread of neural excitation (SOE). Unwanted overlaps of excitation may change the efficiency of subsequent stimuli, or, if sufficient, it can trigger the firing of neighboring neurons, which leads to poor vowel and consonant recognition and poor auditory rehabilitation [67]. Even though current CIs have 12–22 electrodes and channels, due to channel interactions, the number of independent channels is smaller than the actual number of activated electrodes [66]. Although interactions may occur between non-adjacent electrodes, they are greatest and strongest between adjacent electrodes [66,69]. For better hearing, larger interelectrode spacing can be used to reduce channel interactions between adjacent electrodes [19]. Studies have shown that improved spectral resolution and better speech understanding are achievable with lesser channel interactions [70,71,72].

**Figure 4:**
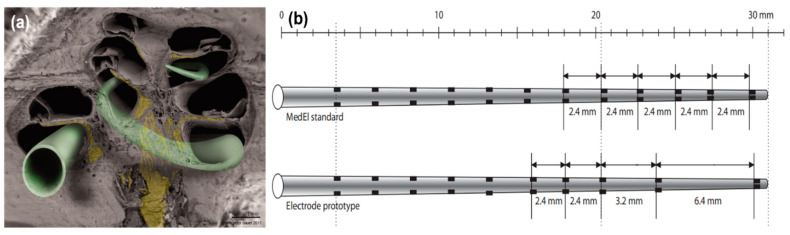
(**a**) Scanning electron microscopy (SEM) image of a hemisectioned human cochlea. Neuronal components are shown in yellow. The electrode array in green indicates potential electrode positions. Some electrode designs target the electrode array to a site close to the modiolus (perimodiolar-lower basal turn at the left lower corner of the figure), while non–preshaped electrodes are placed closer to the spiral ligament and osseous spiral lamina (other electrode positions). Reprinted with permission from [73]. Copyright 2012, Wiley. (**b**) A standard MedEl electrode array (with a length of 26.4 mm) that contains 12 electrodes. The interelectrode spacing is 2.4 mm. A prototype electrode array (with a length of 26.4 mm) that contains 10 electrodes. The interelectrode spacing is 2.4 mm to 3.2 mm in the basal and middle part and 6.4 mm in the middle and apical part of the electrode array. Reprinted with permission from [69], Copyright 2007, Karger.

The anatomical structure of the cochlea makes it challenging to produce cochlear implants. The main reason for this is the narrowing of the width of the cochlea towards the apex region [73]. As depicted by the colored scanning electron microscope (SEM) image of the cochlea shown in Figure 4a, this narrowing is clearly visible, and it limits the insertion of the electrode array into the apex region. A new electrode array with a larger interspacing of the electrode is shown in Figure 4b. Due to strong channel interactions in the apical cochlea and the deep electrode insertion for optimal performance, a new design was proposed, which helped to demonstrate that larger interspacing of electrodes in the apical part decreased channel interactions and increased speech perception abilities [69].

**Figure 5:**
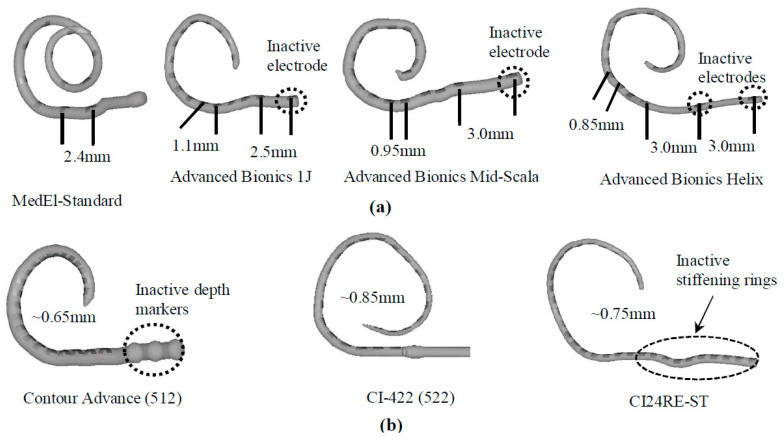
Electrode spacing of seven major types of cochlear implant (CI) electrode arrays. Distantly (**a**) and closely (**b**) spaced electrode arrays. Reprinted with permissions from [75,76]. Copyright 2019, with permission from Elsevier and from the author, respectively.

Longer electrode arrays with fixed number of channels provide larger interelectrode spacing and reduce channel interaction. Although deeper insertion with a long electrode array enhances the range of place pitch coding, perceptual distances between adjacent electrodes are likely to decrease in the apex. To compensate for this, an electrode array could be designed such that spacing between the electrodes in the apex is greater than the spacing between the electrodes in the rest of the array [74]. Interelectrode spacing can be classified into two groups, distantly-spaced and closely-spaced [75]. Table 1 and Figure 5 summarize the interelectrode spacings of CIs produced by different manufacturers [75,76].

### 2.4. Electrode Arrays

There are three types of electrode arrays, perimodiolar, lateral wall, and mid-scala. Perimodiolar electrode arrays, also called modiolus-hugging or counter type arrays, are placed close to the modiolus wall [77]. In this configuration, the electrode array is placed as near as possible to the modiolus, which contains spiral ganglion cells, resulting in the generation of lower electrical thresholds for stimulation, higher dynamic ranges, and less channel interaction as compared to normal implant electrodes, which are usually located peripherally within the scala tympani [36]. Lateral wall electrode arrays are placed along the lateral wall of the scala tympani. All lateral wall electrode arrays face the lateral wall at an angle of approximately 180°, which may lead to trauma of the scala tympani [78]. The perimodiolar type offers advantages over the lateral wall type in terms of lower stimulation levels, an expanded dynamic range, and better channel separation. Because perimodiolar configuration provides a placement of electrodes closer to spiral ganglions than the lateral wall, it leads to better neural stimulation, which may result in better speech perception [79]. Both lateral wall and perimodiolar electrode arrays can cause intra-cochlear trauma, but perimodiolar electrode arrays are likely to deviate to the scala vestibuli from the scala tympani more often than the lateral wall electrode arrays, leading to damage of the osseous spiral lamina/spiral ligament, which can induce new bone formation and ultimately impact the hearing quality [77]. Mid-scala electrode arrays are implanted in the middle of the scala tympani, where this design minimizes trauma to the modiolus wall or lateral wall, albeit with a lower quality of stimulation [80].

### 2.5. Stiffness and Flexibility of Electrode Arrays

During the insertion of CI electrode arrays, different ranges of trauma may occur at the delicate anatomical regions of the cochlea such as the basilar membrane, osseous spiral lamina, scala tympani, and scala vestibuli. Intracochlear trauma may cause severe damage to the dendrites, spiral ganglion cells, and the specific distribution of these cells, and it can also result in the efficiency of the stimulation of sites located along the implant electrodes and a loss of high-fidelity sound [9,81]. It was found that 10–30% of patients lose their residual hearing after CI insertion [82]. Properties such as size, dimensions, and stiffness are important factors for the insertion and final positioning of electrode arrays within the scala tympani. Specifically, stiffness of electrode arrays is significantly related to the trauma to cochlear structures [9,81,83,84,85,86]. For example, if the electrode array tip is very stiff, it could penetrate the spiral ligament during insertion, and the surgeon may not feel the pressure [77]. The stiffness of electrode arrays is, therefore, an important factor for the effective function of CI devices. There are many factors affecting the stiffness of the electrode array, such as the size and thickness of the electrode contact pad, wire thickness and electrode material (i.e., Pt, and Pt–Ir alloy), insulating material around wires (i.e., Teflon, Parylene, and silicon), and the number of individual stimulating channels [77].

**Figure 6:**
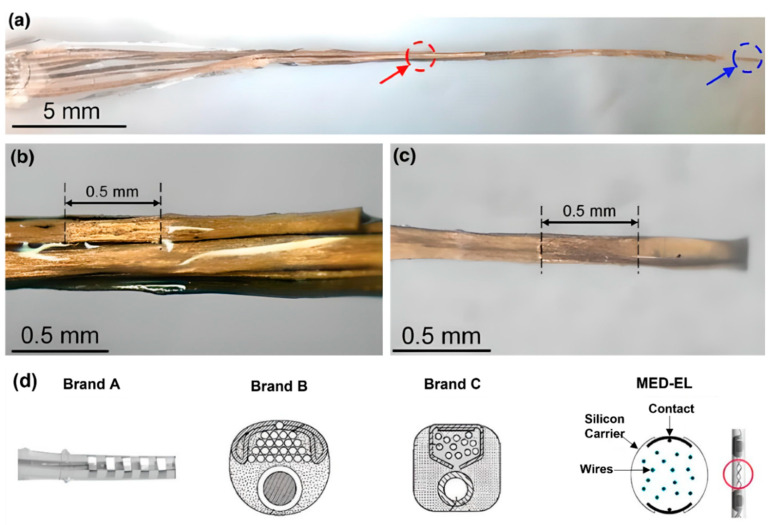
(**a**–**c**) Carbon nanotube (CNT) bundle-based intracochlear electrode array. (**a**) Red circle indicates stimulation site at the base, and blue circle indicates stimulation site at the apex of the CI. A closer photograph of the stimulation site (**b**) at the base, (**c**) at the apex. Reprinted with permission from [87]. Copyright 2019, Springer Nature. (**d**) Straight and wavy wire management/distribution of electrode arrays by brand A, B, C, and MED-EL. Reprinted with permission from [77]. Copyright 2017, Elsevier.

Wire bundling also determines the stiffness of the device. Conventional CIs contain Pt or Pt–Ir wires, which increase the stiffness of the electrode array. An electrode array with carbon nanotube (CNT) bundles instead of metal wires, where eight CNT bundles were coated with Parylene-C for insulation, was developed (Figure 6a–c) [87]. After coating, each CNT bundle was encapsulated with a silicone elastomer. Developed electrode array had a thickness of 135 µm at the apex and 395 µm at the base. These dimensions are smaller than those of conventional intracochlear electrode arrays. Thin and flexible CNT bundle-based electrode arrays required a sixfold lower force for insertion and extraction than metal wire-based intracochlear electrode arrays, reducing the risk of trauma [87]. While some CI manufacturers prefer straight wires, MED-EL uses wavy wires which spread forces and prevent electrodes from acting like needles that can cause damage during the insertion (Figure 6d) [77].

**Figure 7:**
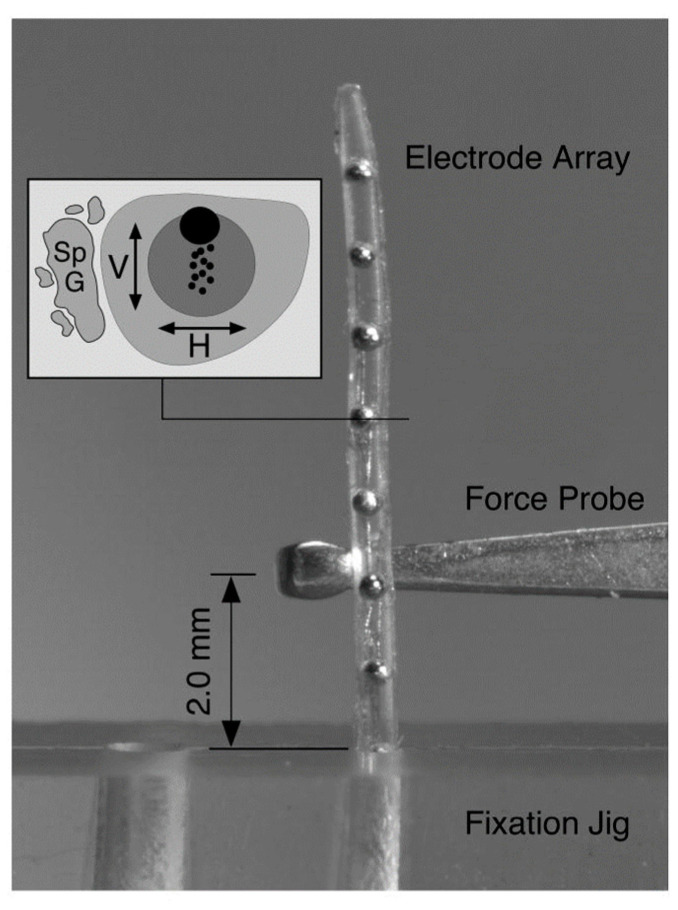
Deflection force required to bend each electrode array 30° at a distance of 2 mm from the fulcrum of a fixation jig that was measured with a mechanical force gauge at 1 mm intervals across the length of each electrode. The stiffness of the electrode array was deducted from the experiment. Reprinted with permission from [9]. Copyright 2008, United States Department of Veterans Affairs.

Rebscher et al. studied different kinds of electrode arrays produced by Cochlear Limited, Advanced Bionics, and Nurobiosys. In this study, electrode arrays were bent at the horizontal and vertical planes with a deflection force (Figure 7), and it was found that the risk of penetration to cochlear structures with stiffer electrode arrays in the vertical plane is lower than that of electrode arrays that have isotropic or higher stiffness in the horizontal plane [9]. Another study characterized the stiffnesses of the Nucleus straight and contour electrode arrays along their length, and it was reported that the Nucleus straight array has a Young’s modulus of elasticity increasing from the tip (182 MPa) to the rear end (491 MPa). Contour array Young’s modulus was greatest at the tip (480 MPa) and almost uniform in the middle and rear end segments (380–400 MPa). Because buckling during electrode insertion may lead to penetration of the basilar membrane by the array tip, buckling experiments were performed, and it was found that contour electrode array had roughly four-times-higher critical buckling load than Nucleus straight array [81].

Biocompatibility, flexibility (Young’s modulus, and elongation to break), and ease of processing are important factors for choosing an electrode array backing substrate. Silicon is commonly used as a backing material [17], because silicon substrates are robust, have out-of-plane flexibility [88], and can sustain rotation and bending [17]. However, if fracture limit is exceeded, silicon-based substrates can break during insertion. The Young’s modulus of silicon is 169 GPa, of silicon nitride is 222 GPa and of silicon dioxide is 70 GPa. Polymers that have a lower Young’s modulus such as Parylene-C (2–4 GPa) or polyimide (~7 GPa) can be used as alternatives to silicon for the fabrication of electrode array [88,89]. Because Parylene-C has a high elongation-to-break value (higher than 200%), low Young’s modulus, Food and Drug Administration (FDA) approval for use in the fabrication of long-term implants, and ability to be easily etched with O_2_ plasma, it stands out as a potential electrode array substrate material [17]. To improve electrode flexibility while maintaining robustness, Parylene-C film was used for coating of somewhat stiff silicon, leading to decreased stiffness of the electrode array by up to 75% [89]. Polymer-based electrode arrays, however, face issues during surgical insertion due to the lack of structural stiffness. To stiffen Parylene-C electrode arrays, Kapton tape was used as an electrode array carrier material for long-term implants. The incorporation of Kapton tape increased the bending stiffness of the Parylene-C array by 60% [90]. In addition to stiffness, the size of electrode arrays affects the flexibility of the implant [91]. The main commercial CI manufacturer models and their electrode array dimensions are given in Table 2 [87,92,93,94,95,96,97,98].

## 3. Modes of Stimulation

**Figure 8:**
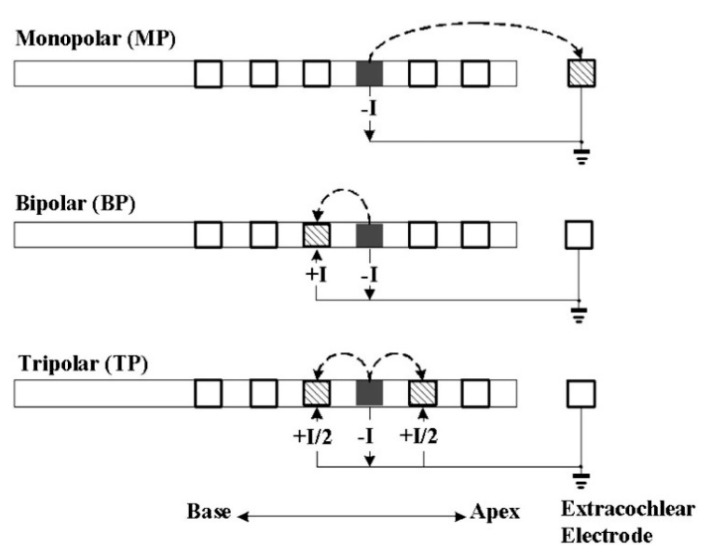
Schematic of different stimulation modes. Monopolar (**top**), bipolar (**middle**), and tripolar (**bottom**) stimulations, all have the same active electrode (gray bar; EL = electrode) but different return electrodes (hatched bars; EL − 1 or EL + 1 or EC = extra-cochlear electrode). Solid lines refer to the electric circuit. Dashed arrow represents current path from the active to return electrode, with (−I) indicating the cathodic phase. Reprinted with permission from [22]. Copyright 2012, Elsevier.

Sharp spatial resolution at the electrodes is critical for high auditory performance. For many years, monopolar stimulation has been preferred in CI electrodes; however, discrimination of human voices was a challenge with monopolar stimulation. Therefore, alternative stimulation approaches such as bipolar and tripolar approaches have been proposed (Figure 8). With different stimulation types, threshold stimulation value, electrical dynamic range, stimulation intensity, and neural curves vary. In monopolar stimulation, which is widely used in clinical practice, electrical current flows between one of the intracochlear electrode contacts and extracochlear reference electrode. Generally, there are two reference electrodes placed on the body of the implant and below the temporal muscle. In case something goes wrong with one of the reference electrodes, the other one completes the monopolar stimulation current circle. To avoid stimulation at the outside of the cochlea and to keep the current density low, the extracochlear reference electrode usually has an at least 10-times-larger surface area than the active electrode [99]. Therefore, the CI performance in monopolar stimulation is affected by the impedance of the intracochlear and extracochlear reference electrodes. In bipolar stimulation, two intracochlear electrodes are stimulated, with opposite polarity and current flows between these active and reference electrodes [100,101]. In tripolar stimulation, two adjacent electrodes function as a reference, with each of them receiving half of the current delivered to the active electrode [22].

Contrary to monopolar stimulation, tripolar stimulates a limited number of spiral ganglion cells, providing a higher spatial resolution. However, there are still doubts about the quality of hearing achieved using tripolar stimulation [22]. To investigate the excitation patterns of different stimulation modes, computational experimental models have been developed. A 3D volume conduction model and an active nerve fiber model were used, and it was concluded that the differences between the spatial excitation models of the various multiples cannot be simulated in a model containing linearly aligned neurons with the same morphology at equal volumes [102]. Similarly, another study reported narrower spatial activity for focused stimulation with the bipolar or tripolar mode than the monopolar stimulus [103]. The success of the CI electrodes in stimulation modes can be determined by tonotopic activity. The nature of the tonotopic activity can be changed by effectively directing current with the time, velocity, and spatial resolution [104]. Recently, studies on the use of partial tripolar stimulation to minimize the negative effects of tripolar stimulation were reported [72]. For example, a computer model of CI stimulation was developed to simulate neural activation in a simplified cochlear geometry [105]. In this study, interactions occurring at the electrode–neuron interface and variations in spiral ganglion nerve density were determined, which can also be used in neural degeneration studies in the future. Surprisingly, virtual channel electrodes proved to be more successful in terms of word recognition and spectral resolution than tripolar and monopolar stimulations [70]. On the other hand, CI applications are more common in pediatric patients than in adult patients. In a study on pediatric CI users, the contribution of electrode interaction to channel independence and implant performance could not be determined [106]. The biggest challenge researchers faced in this regard is the limited datasets. In addition, testing CI electrodes by using different stimulation modes and channel numbers under appropriate conditions also creates a serious limitation. To this end, Saoji et al. solved the problem, albeit partially, by creating a phantom electrode stimulation environment [107]. However, more studies are needed on phantom electrodes to expand the pitch range in cochlear implant receivers, which can provide better coding of the speech spectrum.

## 4. The Use of Sensors with CI Electrode Arrays

CI surgery typically includes a cortical mastoidectomy and posterior tympanotomy to access the middle ear [108]. The cochlea can be entered through the round window or by performing a separate cochleostomy. For the best CI operative efficiency, the electrode should be placed and remain within the scala tympani [33]. Studies have shown that the protrusion of the electrode beyond the scala tympani negatively affects hearing performance [109]. As expected, full electrode placement provides better hearing performance than partial electrode placement [109,110]. Therefore, the ideal surgical procedure would involve a complete insertion of the electrode array into the scala tympani without damaging the basilar membrane or stria vascularis or advancing to adjacent structures. The risk of trauma in CI surgery is high [111]. Therefore, a classification system for cochlear implant insertion trauma has been developed. This divides trauma into four groups: (1) basilar membrane elevation, (2) basilar membrane disruption, (3) disruption of the orbit from the scala tympani to the scala vestibuli, and (4) rupture of the spiral lamina, modiolus, and scala vestibuli (Table 3) [112].

**Figure 9:**
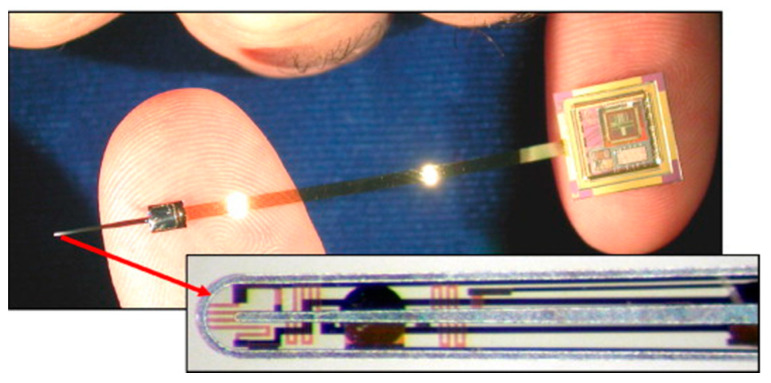
An eight-mm thin film electrode array containing a wall-contact and eight strain gauge sensors at the tip. Reprinted with permission from [113]. Copyright 2008, Elsevier.

Unfortunately, current clinical and commercial practice do not make use of intraoperative force feedback system. In the traditional method, the CI electrode array is still placed by the surgeon who pushes it into the cochlea with additional force. This primitive method continues to be applied for CI devices that have been in use since the 1960s. Therefore, CI designs need to be improved. An internal sensor embedded in the CI electrode array was proposed to measure the contact force and provide real-time feedback to the surgeon on the force applied during the implant placement in the cochlea [76]. Specifically, Bragg grating optical fibers were embedded in the CI electrode arrays, and a contact force reading of 1 mN was demonstrated, which is less than the force required to rupture the cochlea [77]. As an alternative approach, an electrode probe with polycrystalline diamond piezoresistive position sensors for position sensing during implantation was successfully demonstrated [78,79]. A thin-film electrode array containing a wall contact sensor at the tip and eight strain gauge polysilicon piezoresistive sensors in the rest of the electrode array body was fabricated using lithography techniques (Figure 9). Array shape was visualized in real time with a resolution higher than 50 μm during insertion via piezoresistive sensors of thin-film electrode arrays, while tip sensors measured forces on the contacting substrates [80]. An electrode array with integrated position sensors was developed using batch micro processing technique to reduce tissue damage during implant surgery [80]. Polysilicon piezoresistive sensors were used to detect the wall contact and recognize the array shape. The electrode array responds to contact pressures of 0.1 MPa, much lower than that of the Nucleus array (1 MPa), allowing surgeons to control implant placement and reduce the risk of cochlear damage [58]. Overall, the integration of sensors into CI electrode arrays can help minimize the risk of tissue damage, optimize the placement of the electrode arrays, and preserve residual hearing.

## 5. Electrical, Electrochemical, and Mechanical Considerations of CI Electrodes

Although the history of CI is not very long, CIs were soon upgraded from a single-electrode version to a multi-electrode version capable of processing digital signals. Current surface biomaterials used for CI fabrication consist mainly of Pt, silicon, Ti, and ceramics [114]. Pt is the most commonly used material due to its corrosion resistance and high biocompatibility. However, many potential electrode materials are being developed with properties that include an increased mechanical compatibility with neural tissue and a significant increase in charge injection capacity over conventional electrode materials such as Pt [115]. These materials can be coated directly onto the metal substrate of the electrodes. While promising, most of these coatings detach from the metal substrate after prolonged electrical stimulation, resulting in the loss of their electrochemical advantages [116]. On the other hand, the biocompatibility of preferred electrode materials, the density of the electrode regions, and electrochemical and mechanical properties are important design parameters for the production of microelectrode arrays [117]. Most implanted electrodes are made of Pt and/or Ir, as they are electrochemically stable and biocompatible. However, fibrous tissue formation in the cochlea may occur after the surgical placement of the cochlear implant. In addition, it is desired that the electrochemical stability of the electrode surfaces is high, and the impedance is low. To this end, electrode coatings can improve neural interfaces by reducing impedance and by increasing the charge injection limit, which may lead to smaller electrodes and improved specificity [115,118]. Additionally, soft electrode coatings can be made to reduce mechanical stiffness. Electrode coatings may be used for the release of drugs to prevent fibrous tissue or infection that may occur in the cochlea [119]. Coatings are advantageous because they can easily be integrated into existing commercial electrodes. However, preferred electrode designs in CI devices depend on the development of a successful electrode–cellular interface. This communication between electrodes and neurons is related to the electrochemical, electromechanical, and surface energy of the electrode. In these devices, even if the electrode materials selected are highly biocompatible, a minimal inflammatory response may occur. Incompatibility between the mechanical properties of hard metal electrodes and the soft surrounding tissue also causes scars [120]. Soft electrodes, on the other hand, may not be electromechanically stable. The ideal electrode continues to be sought through continuous research and development.

## 6. Current Challenges and Future Directions

Although current CI electrode designs can function properly, they are still far from being ideal. Their placement in the cochlea and their inability to totally cover the human hearing range are among the biggest issues. Due to limited number of electrodes and channels of conventional CIs (12–22), further research on integrating more channels should be carried out [121]. Channel interactions occur when adjacent electrodes are too close to each other; therefore, increasing the number of stimulations has been considered unnecessary, and it has been propounded that using more than eight channels is not effective on speech perception [121]. However, while 3–4 channels are adequate for simple sentence recognition, 30 or more channels are required for difficult speech conditions [69]. The strategy is that, when selecting an electrode configuration, as many electrodes as possible should be kept active but not overlapping [59]. Recent research asserted that a higher speech perception score was achieved with more than eight channels [122,123]. Producing high density CI electrode arrays using conventional methods is limited due to channel interactions, reduction in the production yield, and increasing costs [121]. Therefore, innovations are needed for improving CI electrode array designs with a low cost and a high density of electrodes [121].

For neural stimulation, a better spatial resolution can be achieved with smaller electrodes, and it reduces tissue damage. Additionally, any electrode shape can easily be produced with microfabrication [124]. In this regard, it is expected that, similar to neural electrodes, future CIs will be produced in smaller sizes and with more electrodes. Because the shrinking of electrode surface creates a more limited electric field and prevents the interaction with other electrodes, unwanted stimulation would be prevented, and noisy hearing would be resolved. It was found that electrochemical impedance and charge transfer capacity are affected by the electrode array material and the morphology of neural electrodes [124]. In this case, the impedance and charge transfer capacity of smaller CI electrodes can be tailored by electrode materials.

Traumatic electrode placement is also another main challenge. The incorporation of suitable flexible materials and shape memory polymers or alloys into electrode arrays may result in improved results in surgical procedures and provide better hearing. Current electrode arrays cannot enter the narrowest region of the cochlea, apex, which corresponds to 20–500 Hz of the auditory region. Therefore, much thinner electrode wires with adjustable spatial placements may enable substantially improved hearing quality. We also foresee that future CIs will have more mid-modiolar designs, because electrodes that are placed closer to the spiral ganglion cells lead to lower current consumption and better stimulation.

## 7. Conclusions

During the past two decades, CI electrode arrays have significantly developed, with newer designs offering atraumatic insertion, higher number of electrodes, flexible electrodes made up of shape memory alloys, and better stimulation methods. Despite substantial improvements, many challenges are yet to be addressed. One of the key factors affecting high-quality hearing with CIs is the ability to stimulate as many cochlear spiral ganglion cells as possible. However, the current designs of electrode arrays and complex anatomy of the human cochlea do not allow for such stimulation, resulting in noisy and insufficient hearing experience due to the overlapping of electric fields. To eliminate the effect of channel interactions, improvements in the electrode array designs are needed. Another important factor that may affect electrode arrays is the insertion techniques. During the surgical insertion of the implant, trauma to cochlear structures may occur. The type of electrode arrays and the stiffness and hand skills of the surgeon determine the risk of injury during insertion, which directly affect implant performance. In the future, electrode arrays should: (1) reach and stimulate all spiral ganglion cells with less channel interaction, to provide better hearing performance, (2) have enough stiffness and flexibility to enable insertion without tissue damage and minimal trauma, (3) contain novel electrode geometries to stimulate spiral ganglion cells with less power consumption, (4) be biocompatible for long-time use, (5) be patient-specific due to the anatomical variations between different patients, and (6) have real-time traceability during surgical insertion to prevent trauma and ensure correct placement.

## Figures and Tables

**Table 1:** United States Food and Drug Administration (FDA)-approved electrode arrays with the interelectrode spacing and number of electrodes.

Type	Electrode Array Brand	Total Number of Electrodes	Electrode Spacing (Distance in mm)
**Distantly spaced**	Med-El standard	12	2.4
	Med-El Flex28	12	2.1
	Advanced Bionics 1J	17 (1 inactive electrode)	1.1 and 2.5
	Advanced Bionics Mid-Scala	17 (1 inactive electrode)	0.95 and 3.0
	Advanced Bionics Helix	18 (2 inactive electrodes)	0.85 and 3.0
**Closely spaced**	Cochlear Contour Advance	22	~0.65
	Cochlear CI-422	22	~0.90
	Cochlear CI24RE-Straight	32 (10 stiffenning rings)	~0.75

**Table 2:** Manufacturers and dimensions of cochlear implant electrode arrays.

Manufacturer	Electrode	Apex Width (mm)	Base Width (mm)	Length (mm)
Adv. Bionics	HiFocus Mid-Scala	0.5	0.7	18.5
Adv. Bionics	HiFocus SlimJ	0.5/0.26	0.76/0.56	-
Adv. Bionics	HiFocus 1j	0.4/0.6	0.8	25
MED-EL	Flex (20,24,26)	0.5/0.3	0.8	20, 24, 26
MED-EL	Flex 28	0.5/0.4	0.8	28
MED-EL	Flex Soft	0.5/0.4	1.3	31.5
MED-EL	Form (19,24)	0.5	0.8	19, 24
MED-EL	Standard	0.5	1.3	31.5
MED-EL	Medium	0.5	0.8	24
MED-EL	Compressed	0.5	0.7	15
Cochlear	Contour Advance	0.5	0.8	-
Cochlear	Slim Straight	0.3	0.6	-
Cochlear	Nucleus CI24RE Contour Advance	0.5	0.8	20
Cochlear	Nucleus CI624 Slim 20	0.3	0.6	20
Cochlear	Nucleus CI632 Slim Modiolar	0.35	0.475	18.4
Nurobiosys	Nurobiosys	0.5	0.7	-
Oticon	Neuro Zti^CLA^	0.5	1.07	26
Oticon	Neuro Zti^EVO^	0.4	0.5	25
Oticon	Digisonic^®^ SP	0.5	1.07	26
Oticon	Digisonic^®^ SP EVO	0.4	0.5	25

**Table 3:** Classification for cochlear implant insertion traumas.

Specimen No.	Insertion Site	Electrode Array	Catheter in Place for Histologic Study	Cochlear Damage (Grade)	Depth of Insertion of the Electrode Array (Degrees)
1	RW	Flex^Eas^	No	0	5 × 90 = 450
2	RW	Flex^Soft^	No	3	9 × 90 = 810
3	RW	Flex^Eas^	No	0	4 × 90 = 360
4	RW	Flex^Soft^	No	0	5 × 90 = 450
5	Cochleostomy	Flex^Eas^	No	0	4 × 90 = 360
6	Cochleostomy	Flex^Soft^	No	1	7 × 90 = 630
7	Cochleostomy	Flex^Soft^	No	0	7 × 90 = 630
8	Cochleostomy	Flex^Eas^	No	1	4 × 90 = 360
9	RW	None	No	0	None
10	RW	None	No	0	None
11	RW	None	No	0	None
12	RW	None	No	0	None
13	RW	Flex^Soft^	No	0	7 × 90 = 630
14	RW	Flex^Eas^	No	0	4 × 90 = 360
15	RW	None	Yes	0	None

Note: Evaluation was made according to the scale of 0 to 4 published by Eshraghi et al., in which Grade 0 means no observable trauma; Grade 1, elevation of the basilar membrane; Grade 2, rupture of the basilar membrane; Grade 3, electrode array in the scala vestibuli; and Grade 4, severe trauma such as a fracture of the osseous spiral lamina or modiolus or a tear of the stria vascularis. RW indicates round window. Reprinted with permission form [112]. Copyright 2003, Wiley.
